# For Services Rendered

**Published:** 1875-08

**Authors:** 


### FOR SERVICES RENDERED.

Every day of our lives we get letters thanking us for services rendered. Hundreds, possibly thousands, have reached us containing grateful expressions for our endorsement of McComber's beautiful system of constructing boots. Our mere mention of Dr. Mattson's cure for piles, has brought us a number of letters of enquiry and thanks, as has our allusion to the use of Packer's Tar Soap. The article on the new and excellent food—Granulated Wheat Biscuit—has attracted great attention and has brought the Health Food Company, No. 137 Eighth street, New York, more orders than it has been able to fill, though the ovens are running night and day. So of the Zero Refrigerator, the Young America Printing Press, and many other matters which we have investigated for the benefit of our great family of thoughtful readers. As for the Van Deren perscription for bowel diseases in children, we have been fairly overrun with letters and calls concerning it. By and by we shall be able to give a startling report concerning the vast good which these little pellets have done.

But, strange as it has appeared to us, our allusions to the Great Furniture Estabment of DeGraffe & Cochrane, No. 152 West 23d street, has made new friends for our Magazine, and has induced spontaneous expressions of satisfaction from a large number of housekeepers. One gentleman, long an officer of one of our great railroads, called to tell us that the above-named dealers had furnished his house, after he and his wife had gone the rounds of the manufacturers of New York. Observing our allusion to DeGraffe & Cochrane three months ago, this gentleman remarked to his wife——. "Come ! we have been nearly everywhere in our search for suitable furniture at satisfactory prices ; let us now call on this party of whom HALL 'S JOURNAL —which has not deceived us in all these years—speaks so favorably." So the call was made and the purchase followed. Our friend and subscriber, after thanking us for the hint which had sent him to just the right place," courteously invited us to call at his home and judge for ourself—an invitation which we thankfully accepted and which we shall duly honor, by and by. This, not because we have any doubt of the excellence of the articles with which his fine house is supplied, but because we appreciate his attention and shall be rendered happy by a glance at the forms of beauty and symmetry and grace, in wood and upholstry, which the artists on Twenty-third street know so well how to create.

